# Unveiling hidden heterogeneity and inequalities in the continuum of care for reproductive, maternal, and child health services in sub-Saharan Africa: A multilevel latent class analysis approach

**DOI:** 10.1016/j.gloepi.2025.100237

**Published:** 2025-12-11

**Authors:** Abebew Aklog Asmare, Awoke Seyoum Tegegne, Denekew Bitew Belay

**Affiliations:** a Department of Statistics, College of Science, Bahir Dar University, P.O. Box 79, Bahir Dar, Ethiopia; b Department of Statistics, College of Natural and Computational Science, Mekdela Amba University, P.O. Box 32, Tuluawlyia, Ethiopia; c School of Health Systems and Public Health, Faculty of Health Science, University of Pretoria, Pretoria, South Africa

**Keywords:** Inequalities, Multilevel latent class analysis, Optimal utilizers, Posterior class membership, Suboptimal utilizers, Sub-Saharan Africa

## Abstract

Improving reproductive, maternal, newborn, and child health (RMNCH) services is vital for achieving the Sustainable Development Goals (SDGs) for maternal and child survival. This study utilized multilevel latent class analysis (MLCA) on Demographic and Health Surveys (DHS) data from 29 sub-Saharan African (sSA) countries to identify RMNCH service utilization patterns, examine covariate effects, and assess coverage inequalities. Secondary data from the most recent DHS conducted in 29 sSA countries from 2015 to 2024 were used. MLCA was performed on 12 RMNCH service indicators to account for the hierarchical structure of the data. Summary inequality indicators were used to assess differences in posterior class membership for lower-level classes across wealth quintiles, maternal education, maternal occupation, and place of residence. Women's RMNCH service utilization was divided into two categories: optimal and suboptimal users, and two higher-level categories: high and low coverage. Higher maternal education, household wealth, media access, and early antenatal care were related to a higher likelihood of being in the optimal utilizer class. In contrast, rural location and a longer distance to health services were associated with a lower likelihood. Inequality indices revealed significant differences among optimal utilizers, particularly in terms of mother education and household wealth. Targeted interventions are urgently required to promote RMNCH service utilization in sSA by addressing persistent socioeconomic disparities, particularly among women with no education, lower incomes, and low access to health care.

## Highlights

• MLCA classified women into two groups based on their high and low coverage: optimal and substandard RMNCH users.
• Twelve RMNCH indicators were utilized for child immunization, family planning, postnatal care, and pregnancy.
• Stratified by maternal, household, and geographic factors, service utilization varied both within and between countries.
• By identifying populations and regions with low RMNCH use, classification offers evidence for targeted policy.

## Introduction

Improving RMNCH services is critical to meeting the SDGs, notably the targets for maternal and child survival [1]. Although there has been significant global progress in reducing unnecessary fatalities, regional achievements vary [1]. Between 2000 and 2020, the global maternal mortality ratio (MMR) decreased from 339 to 223 deaths per 100,000 live births; however, sSA continues to bear the greatest burden, with an average MMR of 545, more than three times the global average and more than ten times that of Europe and Central Asia [2,3]. Similarly, while under-five mortality has decreased from 76 to 37 deaths per 1000 live births worldwide, children in sSA continue to face the worst risks, with mortality rates roughly five times greater than those in high-income countries [2,3]. These inequalities highlight the need for equity-focused monitoring of RMNCH services.

Despite global promises, access to critical RMNCH services, such as prenatal care, skilled delivery attendance, postnatal care, family planning, and child immunization, remains severely unequal in sSA [4,5]. Women and children in rural areas, poorer households, and those with no education face systematic disadvantages. National averages frequently hide these disparities, implying greater growth than is actually the case [6]. Traditional monitoring methods, such as single coverage indicators and composite indices like the Composite Coverage Index (CCI), are valuable benchmarks, but they oversimplify complex care-seeking behaviors. In some studies, women who scored more than 50 % on the CCI were considered optimal users [7], while others defined higher coverage based on the number of RMNCH service components received [8,9]. Such threshold-based methods impose arbitrary cut-offs that may obscure underlying heterogeneity and misrepresent care patterns.

To the best of our knowledge, no prior research has used methods that encompass the entire continuum of care to investigate RMNCH utilization of services across several sSA countries. The majority of earlier research either employed composite indices like the CCI [7,10,11] or relied on arbitrary thresholds to classify women as higher- or lower-level users [8,9]. These techniques limit their capacity to identify underlying variability because they presume consistent service patterns and may miss significant correlations between individual services. By analyzing patterns of RMNCH service usage across individual- and national-level characteristics, our study fills this gap by offering a more comprehensive picture of consumption practices and revealing disparities across important socioeconomic and demographic categories.

In this study, we examined RMNCH service consumption in 29 sSA nations while taking individual and national characteristics into account. We were able to investigate the relationship between patterns of optimal and suboptimal service usage and factors like maternal education, household socioeconomic position, and national-level health and economic variables. Our analysis emphasizes disparities among various demographics and offers a more thorough knowledge of service consumption by incorporating these variances. In order to understand variation within and between countries, this study employs a novel approach that combines multilevel latent class analysis with inequality assessment, classifying women into latent subgroups based on their RMNCH service utilization and examining disparities across key socioeconomic and demographic dimensions [11,12]. This two-step approach uncovers hidden patterns in service use and connects them to indicators of inequality. This study's primary goals were to find unique patterns in the use of RMNCH services, determine how factors at the individual and national levels affect class membership, and analyze disparities in service coverage across important socioeconomic and demographic dimensions.

## Methods

### Data source and study design

In this study, cross-sectional data from 29 sSA countries' DHS were examined. An MLCA was used to capture country-level variability and account for both fixed and random effects due to the hierarchical structure with mothers nested inside countries.

### Data source and sampling method

Data were drawn from the most recent DHS conducted between 2015 and 2024 in 29 countries: Angola, Benin, Burkina Faso, Cameroon, Chad, Côte d'Ivoire, Democratic Republic of Congo, Ethiopia, Gambia, Gabon, Ghana, Guinea, Kenya, Liberia, Madagascar, Malawi, Mali, Mauritania, Mozambique, Nigeria, Rwanda, Senegal, Sierra Leone, Tanzania, Uganda, Zambia, and Zimbabwe. The DHS used a stratified two-stage cluster sampling design, ensuring national representativeness. Survey data were collected via face-to-face interviews, and child vaccination and maternal care information were self-reported, with recall periods of up to five years. While participation and response rates vary across countries, all surveys followed standardized DHS protocols to ensure data quality and reliability. The DHS Kids Record (KR) file was used to extract child-related information (https://dhsprogram.com/Data/terms-of-use.cfm). Country-level covariates were obtained from the World Bank's World Development Indicators database (https://databank.worldbank.org/source/world-development-indicators).

### Study population

**Fig. 1:**
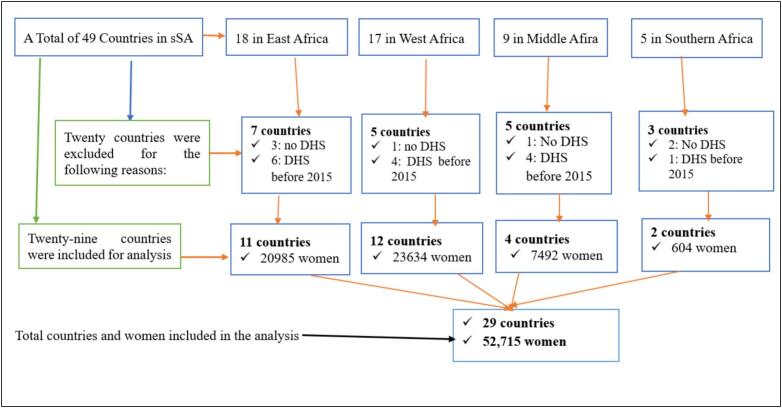
Participant selection flowchart for analysis based on recent DHS reports (2015–2024).

**Fig. 2:**
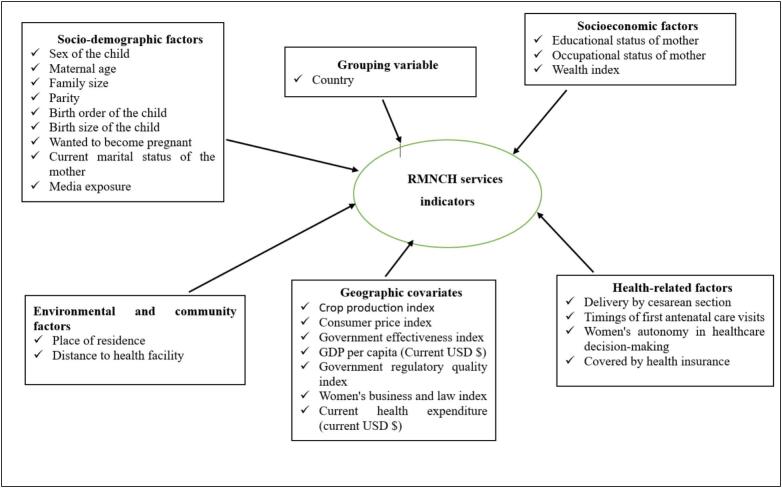
Conceptual framework of individual, household, and higher-level covariates in RMNCH service utilization.

BCG, polio, DPT (diphtheria, pertussis, and tetanus), and measles vaccinations should be administered to children before 12 months of age in accordance with the Expanded Program for Immunization guidelines [13]. We used the children's record (KR) files to concentrate on children between the ages of 12 and 23 months because the DHS records data into distinct datasets [14]. 52,715 women with their most recent kid (alive and living with the mother) between the ages of 12 and 23 months were eligible out of 337,480 women with children under five in the five years before the survey. Fig. 2 shows the nation selection and total sample involved, while Table 2 summarizes the included countries and survey years. (See Fig. 1.)

### Study variables

#### Latent class indicators

This analysis used twelve binary DHS indicators (1 = received, 0 = not received) to describe the RMNCH service continuum, with latent classes representing unobserved coverage patterns. Indicators were harmonized with international standards and updated to reflect changes in the DHS measurements over time. All twelve variables, chosen based on previous research [10,11,15,16], were consistently available in the 29 sSA countries investigated.

Antenatal care was evaluated using check-ups and tetanus toxoid injections, with at least four visits before 2017 and eight visits after 2017, according to WHO guidelines, and at least two doses of tetanus toxoid defined protection against neonatal tetanus. Birth care entailed facility delivery and competent birth attendance by qualified health professionals, while postnatal care included maternal and child evaluations within two days following delivery. Age-appropriate breastfeeding and childhood immunizations (BCG, DPT, polio, and measles) were included, as was maternal use of modern family planning methods. In the MLCA framework, these variables were combined to establish latent categories of service consumption while taking into consideration individual clustering within nations and the hierarchical survey design.

#### Lower and higher-level covariates

Individual and household factors were included to estimate latent class membership in RMNCH service utilization [[7], [8], [9], [10], [11]], while contextual and macro-level factors captured economic, governance, and health system influences [[17], [18], [19], [20], [21]], accounting for within- and between-country variation. The variables included in the analysis are summarized in Fig. 2.

As country-level variables, a variety of indices and economic metrics that capture the structural and policy context were included. Cross-country comparisons of agricultural performance are made possible by the production growth rate, which shows total agricultural output in relation to a base year (base year = 100). An indicator of economic stability, the annual inflation rate monitors shifts in the average level of prices for goods and services. While the Government Regulatory Quality Index measures the perceived capacity of governments to create and carry out sound policies, the Government Effectiveness Index measures perceptions of the quality of public services, civil service capacity, and policy formulation. Both indices are comparable across nations and range from −2.5 to +2.5. The average economic output per person is measured by the GDP per capita (current USD), whereas the total resources allotted to healthcare at the individual level are represented by the current health expenditure per capita (current USD). Gender equality in legal and regulatory frameworks is evaluated using the Women, Business, and the Law (WBL) Index, which has a score range of 0 to 100. Higher scores indicate greater equality. Lastly, the Annual Crop Production Growth Rate was added to account for variations in agricultural production from year to year. Before being included in the multilevel latent class analysis, all variables were normalized to guarantee consistent interpretation and comparability.

### Statistical analysis

We employed MLCA to discover unobserved patterns of RMNCH service utilization in 29 sSA countries. The analysis used data on 52,715 mother–child pairs, capturing receipt of essential RMNCH services across the continuum of care. Lower-level units (women) are organized within higher-level units (countries), allowing MLCA to account for both within- and between-country variability in latent class membership. The posterior probability for latent class assignment was computed, and equity studies looked at variations in class membership based on household wealth, mother's education, urban-rural domicile, and maternal occupation [22,23]. This technique accounts for both within-country and between-country heterogeneity in latent class membership distributions.

Lower-level latent classes ($C_{i}$, k=1,…,K) represent subgroups of women with distinct RMNCH service utilization patterns, while higher-level classes ($W_{j}$, m=1,…,M) capture country-level heterogeneity in the distribution of lower-level classes [24,25]. The probability of observing the RMNCH indicator vector $y_{\mathrm{ij}}={\left(y_{\mathrm{ij}1},\ldots ,y_{\mathrm{ijH}}\right)}^{\prime }\;$for individual iin country j, conditional on higher-level ($Z_{1j}$) and lower-level ($Z_{2\mathrm{ij}}$) covariates, is defined as:

(1) $$P\left(y_{\mathrm{ij}}/Z_{1j},Z_{2\mathrm{ij}}\right)=\sum_{m=1}^{M}P\left(W_{j}=m/Z_{1j}\right)\sum_{k=1}^{K}P\left(C_{i}=k/W_{j}=m,Z_{2\mathrm{ij}}\right)\prod_{h=1}^{H}P\left(y_{\mathrm{ijh}}/C_{i}=k\right)$$

where $\left(W_{j}=m/Z_{1j}\right)$ and $\mathrm{PP}\left(C_{i}=k/W_{j}=m,Z_{2\mathrm{ij}}\right)$ are modeled via logistic regression at the country and individual levels, respectively, and $P\left(y_{\mathrm{ijh}}/C_{i}=k\right)$ denotes indicator probabilities.

Latent class regression linked class membership to covariates [22,26]. Higher-level logistic models are:

(2) $$P\left(W_{j}=m/Z_{j}^{H}\right)=\frac{\exp\left(\alpha_{m}^{\prime }Z_{j}^{H}\right)}{1+\sum_{l=2}^{M}\exp{\left(\alpha_{l}^{\prime }Z_{j}^{H}\right)}^{\prime }}$$

and lower-level models are:

(3) $$P\left(C_{i}=k/W_{j}=m,Z_{\mathrm{ij}}\right)=\frac{\exp\left(\gamma_{\mathrm{km}}^{\prime }Z_{\mathrm{ij}}\right)}{1+\sum_{s=2}^{K}\exp{\left(\gamma_{\mathrm{sm}}^{\prime }Z_{\mathrm{ij}}\right)}^{\prime }}$$

where $\alpha_{m}$ and $\gamma_{\mathrm{km}}\;$are regression coefficients. A two-step estimation approach from the R package ***multilevelLCA*** [27] was employed: first, the measurement model was estimated without covariates; second, structural multinomial regressions linked latent classes to covariates while holding measurement parameters fixed.

Class separation quality was assessed using entropy, computed from posterior probabilities for lower- and higher-level classes:

(4) $$E_{\mathrm{low}}=1-\frac{-\sum_{i=1}^{n}\sum_{k=1}^{K}P_{\mathrm{ik}}\log\left(P_{\mathrm{ik}}\right)}{\mathrm{nlog}\left(K\right)}\;\text{and}\;E_{\mathrm{High}}=1-\frac{-\sum_{i=1}^{n}\sum_{m=1}^{M}P_{\mathrm{im}}\log\left(P_{\mathrm{im}}\right)}{\mathrm{nlog}\left(M\right)}$$

where $P_{\mathrm{ik}}$and $P_{\mathrm{jm}}$are posterior class probabilities. The optimal numbers of lower (K) and higher-level (M) classes were selected sequentially using BIC, AIC, and entropy-adjusted ICL-BIC, with reselection to ensure theoretical interpretability [22].

### Assessment of health inequalities

We utilized the WHO Health Equity Assessment Toolkit (HEAT) framework [28], which outlines standard equity dimensions and recommended tools, to measure socioeconomic and demographic disparities in RMNCH service utilization. In this study, four major dimensions were used to determine inequality: wealth quintiles, maternal employment status, place of residence, and maternal education level. We calculated four kinds of inequality measures using the latent class assignments of individuals into optimal versus suboptimal RMNCH service use obtained from the multilevel latent class analysis: (1) Simple measures (absolute differences and relative ratios); (2) disproportionality measures (absolute and relative concentration indices) for ordered stratifiers; (3) regression-based measures (slope index of inequality [SII] and relative index of inequality [RII] that capture disparities throughout the entire distribution of ordered stratifiers; (4) and impact measures (population attributable risk [PAR] and population attributable fraction [PAF] that quantify the contribution of inequalities to total RMNCH utilization. To produce reliable 95 % confidence intervals, all analyses considered clustering at the national level. The **healthequal** R package [29] was used to perform the calculations. It provides both absolute and relative indices that capture the magnitude and proportional differences in service utilization across population subgroups, guaranteeing that the results reflect actual coverage disparities rather than model coefficients.

### Missing value treatment

In the present study, we applied Multiple Imputation by Chained Equations (MICE) to solve missing data [30,31]. This approach provides a reliable and consistent dataset, which enhances the reliability of our findings [32,33].

### Sensitivity analysis

The 12 RMNCH service components were divided into two conceptually separate categories for sensitivity analyses to evaluate the robustness of the multilevel latent class analysis: maternal care (antenatal care, tetanus toxoid vaccination, skilled birth attendance, facility delivery, postnatal care for mothers and newborns, and family planning) and child care (age-appropriate breastfeeding and childhood vaccinations: BCG, DPT3, polio, and measles) [16]. The consistency and stability of the class structures were assessed by estimating distinct MLCA models for each group and comparing their model fit indices (AIC, BIC-low, and BIC-high) with those from the complete 12 service model. This approach allowed us to verify the robustness of the overall model and determine whether any particular group had a disproportionate influence on the latent class classification.

## Results

The study analyzed DHS data from 29 sSA countries collected between 2015 and 2024, for a total of 52,715 weighted respondents. Sample contributions differed notably by country, with Nigeria contributing the most (10.9 %), followed by the Democratic Republic of the Congo (6.1 %), Kenya (5.1 %), and Malawi. In comparison, South Africa (0.5 %), Lesotho (0.6 %), and Liberia (1.1 %) had the fewest number of respondents. Most countries contributed between 2 % and 5 % of the overall weighted sample (see Table 1).

**Table 1:** Frequency and distribution of percentage for study sample in Sub-Saharan African countries (*n* = 52,715).

Country	DHS Year	Weighted frequency	Percent (%)
Angola	2015/16	1916	3.6
Benin	2017/18	2346	4.5
Burkina Faso	2021	2187	4.1
Burundi	2016/17	2440	4.6
Cameroon	2018	1533	2.9
Côte d'Ivoire	2021	1532	2.9
Democratic Republic of Congo	2023/24	3227	6.1
Ethiopia	2016	1884	3.6
Gabon	2019/21	817	1.5
Gambia	2019/20	1339	2.5
Ghana	2022	1491	2.8
Guinea	2018	1252	2.4
Kenya	2022	2665	5.1
Lesotho	2023/24	342	0.6
Liberia	2019/20	586	1.1
Madagascar	2021	1873	3.6
Malawi	2015/16	2669	5.1
Mali	2023/24	1939	3.7
Mauritania	2019/21	1905	3.6
Mozambique	2022/23	1490	2.8
Nigeria	2018	5771	10.9
Rwanda	2019/20	1341	2.5
Senegal	2023	1807	3.4
Sierra Leone	2018	1480	2.8
South Africa	2016	262	0.5
Tanzania	2022	1793	3.4
Uganda	2016	2324	4.4
Zambia	2018	1490	2.8
Zimbabwe	2015	1016	1.9

### Characteristics of reproductive, maternal, and child health services

**Fig. 3:**
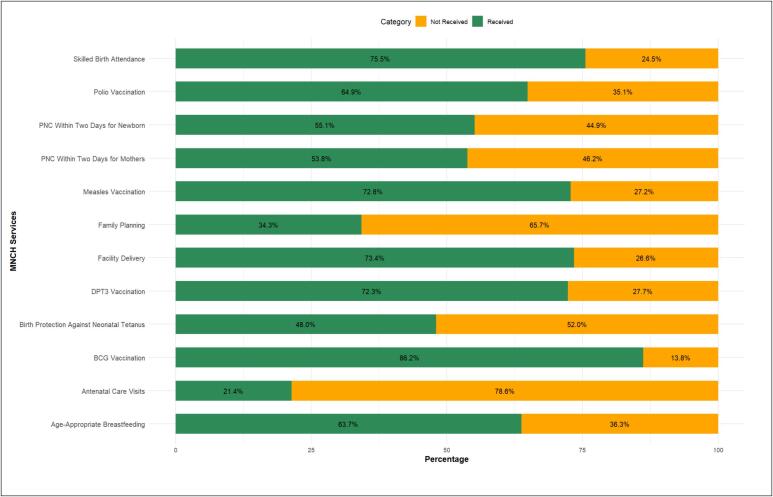
Frequency distributions of RMNCH services.

Among mothers included in the study, 11,270 (21.4 %) had either four or more or eight or more ANC visits, and nearly half of the mothers received two doses of tetanus injection, 25,315 (48.0 %). A total of 38,717 (73.4 %) women delivered in a health facility. For postnatal care, 28,361 (53.8 %) women received a health checkup within two days of delivery, while 29,049 (55.1 %) newborns had a postnatal check within two days. Of them, 20,089 (35.2 %) used modern contraceptive methods. Among the four vaccinations, 45,452 (86.2 %), 38,119 (72.3 %), 38,388 (72.8 %), and 34,210 (64.9 %) women had children who received BCG, three doses of DPT, measles, and three doses of polio, respectively. A total of 33,599 (63.7 %) children received age-appropriate breastfeeding (Fig. 3).

### Characteristics of covariate variables

Table 2 shows the distribution of the major individual-level independent factors. The majority of women (71.8 %) were aged 20–34 years, and 67.2 % lived in rural regions. Most women (90.2 %) lived in families without health insurance. In terms of decision-making autonomy, 58.4 % of women had the freedom to choose their own medical treatment. The most common form of delivery was vaginal, with 93.3 % not having undergone cesarean delivery. In terms of socioeconomic level, approximately 20 % were in the middle wealth quartile. Approximately half of all mothers (49.1 %) had a female child as their most recent child. Early prenatal care was widely used, with 38.4 % of women having their first visit during the first trimester.

**Table 2:** Individual-level independent variable description and frequency distribution in Sub-Saharan Africa countries (*n* = 52,715).

Lower-level predictor variables	Categories	Weighted frequency (%)
Maternal age	Less than 20	3185 (6.0)
	20 to 34	37,862 (71.8)
	35 to 49	11,668 (22.1)
Place of residence	Urban	17,269 (32.8)
	Rural	35,446 (67.2)
Level of maternal education	No education	19,135 (36.3)
	Primary education	17,147 (32.5)
	Secondary education	13,835 (26.2)
	Higher education	2598 (4.9)
Family size	Small	15,310 (29.0)
	Medium	28,739 (54.5)
	Large	8666 (16.4)
Media access	No	19,392 (36.8)
	Yes	33,323 (63.2)
The birth size of the child	Small	8053 (15.3)
	Average	27,371 (51.9)
	Large	17,291 (32.8)
Parity	1 to 4	36,418 (69.1)
	5 and more	16,297 (30.9)
Wanted to become pregnant	No or later	13,720 (26.0)
	Yes	38,994 (74.0)
Distance to health facility	Not a big problem	32,461 (61.6)
	Big problem	20,253 (38.4)
Covered by health insurance	No	47,534 (90.2)
	Yes	5181 (9.8)
Mother occupation	Not working	16,126 (30.6)
	Working	36,589 (69.4)
Women's autonomy in healthcare decision-making	No	21,932 (41.6)
	Yes	30,782 (58.4)
Birth order of the child	First	9929 (18.8)
	2 to 3	19,594 (37.2)
	4 to 5	12,655 (24.0)
	6 and more	10,538 (20.0)
Sex of the child	Male	26,833 (50.9)
	Female	25,882 (49.1)
Timings of first antenatal care visits	First trimester	21,951 (38.4)
	After the first trimester	35,177 (61.6)
Delivery by cesarean section	No	49,157 (93.3)
	Yes	3558 (6.7)
Wealth index	Poor	23,482 (44.5)
	Middle	10,480 (19.9)
	Rich	18,752 (35.6)
Ever terminated pregnancy	No	45,317 (86.0)
	Yes	7504 (14.0)


Lower-level predictor variables	Categories	Weighted frequency (%)
Sex of household head	Male	44,843 (85.1)
	Female	7872 (14.9)
Husband's education level	No education	17,272 (32.8)
	Primary education	15,051 (28.6)
	Secondary education	15,721 (29.6)
	Higher education	4671 (8.9)
Husband occupation	Not working	3818 (7.2)
	Working	48,902 (92.8)
DHS-SDG period	Pre SDG	5601 (10.6)
	Early SDG	28,708 (54.5)
	Action of decades	18,406 (34.9)

### Multilevel latent class regression results

#### Measurement model

The MLCA model distinguished two unique groups of women and two groups of countries based on 12 RMNCH service indicators and child, maternal, household-level, and country-level characteristics. Individually, women were classified as optimal utilizers, who were more likely to receive most RMNCH services across the continuum of care, or suboptimal utilizers, who were less likely to use services. At the country level, countries were classified as higher-coverage countries, where women were more likely to obtain RMNCH services, or lower-coverage countries, where women had reduced chances of service uptake.

Model fit statistics corroborated the two-class approach: for women, the multilevel model had superior classification quality (entropy = 1.00, compared to 0.91 in the single-level model) and high overall fit (BIC = 611,937.65; AIC = 611,377.69) than the single-level model. Similarly, for countries, the multilevel technique provided a high match (${\mathrm{BIC}}_{H}$ = 611,463.82, $\mathrm{ICL}-{\mathrm{BIC}}_{H}$= 611,463.82) than models without higher-level structure. According to model selection criteria (BIC, AIC), no intermediate groups were found, confirming that the two-class solution efficiently captured heterogeneity at both the women and country levels. These classifications are relative and data-driven, indicating patterns of RMNCH service consumption rather than a predefined list of components (see Fig. 5).

High coverage countries made up 65.6 % of the sample, with low coverage countries accounting for 34.4 %. Individually, 69.5 % of women were optimal utilizers, with the remaining 30.5 % being suboptimal utilizers. In countries with high coverage, 82.7 % of women were optimal utilizers, while 17.3 % were suboptimal utilizers. In low-coverage countries, 46.5 % were optimal, and 53.5 % were suboptimal (see Table 3 and Table 4).

**Table 3:** Estimated individual-class proportions given country-level classes.

		Higher level class (country)	
		High coverage	Low coverage
Lower-level class (women)	Optimal utilizers	0.827	0.465
	Suboptimal utilizers	0.173	0.535

**Table 4:** Higher-level and lower-level class proportions.

Low coverage class		High-class	
Class assignment level	% of class level	Class assignment level	% of class level
Optimal utilizers	69.5	High coverage	65.6
Suboptimal utilizers	30.5	Low coverage	34.4

**Fig. 4:**
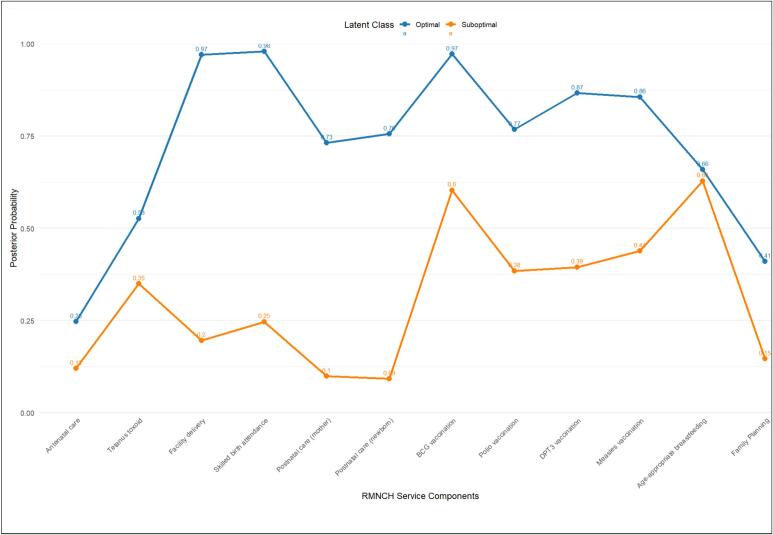
Posterior probabilities of RMNCH services for the two optimal and suboptimal RMNCH service utilization.

Fig. 4 displays the posterior probabilities of individual RMNCH services. Optimal utilizers had higher probabilities than suboptimal utilizers for facility delivery (0.97 versus 0.20), skilled birth attendance (0.98 versus 0.25), postnatal care for mothers (0.73 versus 0.10) and newborns (0.76 versus 0.09), and immunizations: BCG (0.97 versus 0.60), DPT (0.87 versus 0.39), polio (0.77 versus 0.38), and measles (0.86 versus 0.44). Antenatal care (0.25 versus 0.12) and family planning (0.41 versus 0.15) exhibited disparities in uptake, although age-appropriate breastfeeding was comparable across classes (0.66 versus 0.63). These trends reflect diverse service utilization profiles, highlighting the importance of tailored and context-specific public health initiatives.

**Fig. 5:**
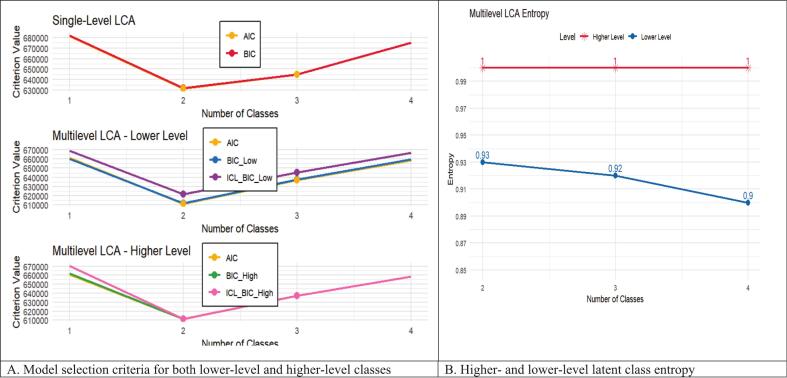
Model selection criteria and latent class entropy for both lower-level and higher-level classes.

**Fig. 6:**
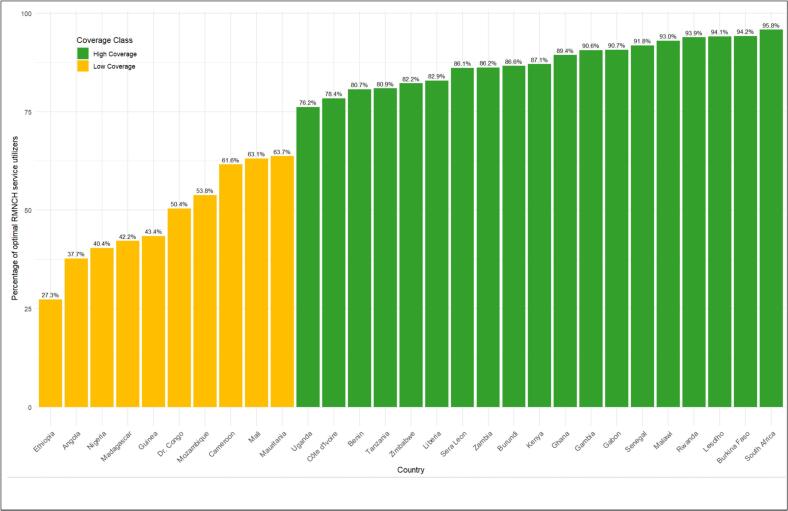
Country assignment to higher-level latent classes based on maximum a posteriori classification.

Fig. 6 and Table 4 reveal that substantial disparities exist in optimal RMNCH service utilization across sSA countries, with coverage ranging from 27.3 % in Ethiopia to 95.8 % in South Africa. Countries were classified into two distinct coverage classes, with a clear separation between low coverage and high coverage groups, highlighting the variability in service uptake across the regions.

### Structural latent class regression model

#### Logistic model for higher-level class membership

To account for variability at the country level, a multilevel logistic regression was employed to predict higher-level latent class membership using country-specific factors. The model indicated meaningful differences in RMNCH service coverage between countries. The higher-level random intercept variance was 1.10 (95 % CI: 1.01–1.61), showing that countries differed in their underlying probability of belonging to the high-coverage class. The intraclass correlation coefficient (ICC) was 25.1 %, meaning that approximately one-quarter of the total variation in class membership was attributable to country-level differences. The median odds ratio (MOR) was 2.76, indicating that the odds of being classified as having high coverage typically differed almost threefold between two randomly selected countries (see Table 5).

**Table 5:** Determinants of high versus low coverage country classification.

Higher-level covariates	AOR (95 % CI)	P -value
Intercept	1.45 (0.84–1.73)	0.139
Annual crop production growth	1.19 (1.15–1.22)	<0.001
Annual inflation rate	0.51 (0.42–0.61)	<0.001
Government effectiveness index	1.23 (1.18–1.29)	<0.001
GDP per capita (current $USD)	1.21 (1.18–1.24)	<0.001
Government regulatory quality index	1.43 (0.97–1.94)	0.134
Women, business, and law index	1.27 (1.24–1.30)	<0.001
Current health expenditure (current $USD)	1.19 (1.18–1.21)	<0.001

Random components		
Community variance	1.10 (1.01–1.61)	
Intraclass correlation (%)	25.06 %	
Median odds ratio	2.76	

Covariates at the country level were linked to membership in the high-coverage RMNCH service class. The likelihood of optimal RMNCH service coverage was positively correlated with annual crop production growth (AOR: 1.19, 95 % CI: 1.15–1.22), government effectiveness (AOR: 1.23, 95 % CI: 1.18–1.29), GDP per capita (AOR: 1.21, 95 % CI: 1.18–1.24), women's business and law index (AOR: 1.27, 95 % CI: 1.24–1.30), and current health expenditure. AOR: 0.51, 95 % CI: 0.42–0.61, the annual inflation rate, was linked to reduced coverage. Government regulatory quality demonstrated a positive correlation (AOR: 1.43, 95 % CI: 0.97–1.94).

Table 6 shows the logistic regression estimates for women's membership in the optimal utilizer class, broken down by the higher-level country class. Adjusted Odds Ratios (AORs) and 95 % confidence intervals are presented for each predictor, illustrating the relationship between individual and household-level attributes and the likelihood of being an Optimal Utilizer within each larger setting.

**Table 6:** Logistic parameter estimates from latent class regression for individual and household level determinants of RMNCH optimal service utilization, stratified by high and low coverage countries.

Lower-level predictor variables	Categories	Frequency of high coverage countries		High coverage countries	
		Suboptimal utilizer	Optimal utilizer	OR (95 % CI)	P -value
Maternal age	Less than 20	221	1297	Ref.	
	20 to 34	2762	19,045	1.09 (0.92–1.29)	0.3
	35 to 49	996	5605	1.17 (0.97–1.42)	0.1
Place of residence	Urban	606	9226	Ref.	
	Rural	3373	16,721	0.96 (0.86–1.06)	0.4
Maternal education	No education	1925	7870	Ref.	
	Primary education	1574	9358	1.08 (0.98–1.18)	0.1
	Secondary education	455	7193	1.13 (1.10–1.28)	0.04
	Higher education	25	1526	1.06 (0.73–1.54)	0.8
Sex of household head	Male	3418	21,699	Ref.	
	Female	561	4248	0.98 (0.89–1.08)	0.7
Wealth index	Poor	2768	10,397	Ref.	
	Middle	656	5185	1.18 (1.07–1.30)	<0.001
	Rich	555	10,366	1.23 (1.09–1.38)	<0.001
Parity	1 to 4	2555	19,285	Ref.	
	5 and more	1724	6662	0.93 (0.82–1.06)	0.3
Evert terminated the pregnancy	No	3409	22,086	Ref.	
	Yes	570	3879	0.98 (0.89–1.08)	0.6
Wanted to become pregnant	No or later	1331	7818	Ref.	
	Yes	2648	18,129	1.01 (0.94–1.09)	0.7
Birth order of the child	First	426	5473	Ref.	
	2 to 3	1326	10,360	0.91 (0.80–1.03)	0.1
	4 to 5	1084	6080	0.89 (0.76–1.04)	0.1
	6 and more	1143	4034	0.82 (0.67–1.01)	0.1
Sex of child	Male	1940	13,230	Ref.	
	Female	2040	12,718	0.98 (0.91–1.05)	0.5
The birth size of the child	Small	629	3786	Ref.	
	Average	2152	13,703	1.02 (0.93–1.13)	0.7
	Large	1198	8459	1.05 (0.94–1.16)	0.4


Lower-level predictor variables	Categories	Frequency of high coverage countries		High coverage countries	
		Suboptimal utilizer	Optimal utilizer	OR (95 % CI)	P -value
Delivery by cesarean section	No	3957	23,304	Ref.	
	Yes	22	2644	1.25 (0.94–1.67)	0.1
Timings of first antenatal care visits	After the first trimester	2652	13,171	Ref.	
	In the first trimester	16,721	12,776	1.15 (1.07–1.23)	<0.001
Women's autonomy in healthcare decision-making	No	1805	9737	Ref.	
	Yes	2174	16,221	1.03 (0.96–1.10)	0.5
Mother occupation	Not working	1315	8133	Ref.	
	Working	2664	17,814	1.04 (0.97–1.13)	0.3
Husband occupation	Not working	441	1596	Ref.	
	Working	3568	24,352	1.13 (1.02–1.27)	0.04
Husband's education level	No education	1762	7553	Ref.	
	Primary education	1493	8273	1.04 (0.95–1.13)	0.4
	Secondary education	643	7594	1.09 (0.97–1.22)	0.1
	Higher education	82	2527	1.07 (0.85–1.35)	0.6
Distance to health facility	Not a big problem	1797	16,803	Ref.	
	Big problem	2182	9145	0.89 (0.83–0.95)	<0.001
Covered by health insurance	No	3659	21,799	Ref.	
	Yes	320	4149	1.03 (0.91–1.15)	0.7
Media access	No	1985	7369	Ref.	
	Yes	1990	18,579	1.12 (1.04–1.22)	0.003
Family size	Small	857	8222	0.98 (0.89–1.08)	0.7
	Medium	2344	13,547	0.97 (0.87–1.09)	0.6
	Large	778	4178	Ref.	
DHS-SDG period	Pre SDG	369	3316	Ref.	
	Early SDG	2141	12,286	1.47 (1.12–1.96)	0.02
	Action of decades	1470	10,345	1.67 (1.10–2.16)	0.02


Lower-level predictor variables	Categories	Frequency of low-coverage countries		Low coverage countries	
		Suboptimal utilizer	Optimal utilizer	OR (95 % CI)	P -value
Maternal age	Less than 20	964	702	Ref.	
	20 to 34	8374	7682	1.11 (0.97–1.28)	0.1
	35 to 49	2738	2329	1.37 (1.16–1.63)	<0.001
Place of residence	Urban	1989	5447	Ref.	
	Rural	10,086	5265	0.81 (0.74–0.88)	<0.001
Maternal education	No education	6777	2562	Ref.	
	Primary education	3425	2790	1.48 (1.36–1.62)	<0.001
	Secondary education	1834	4353	1.90 (1.71–2.11)	<0.001
	Higher education	39	1007	3.25 (2.37–4.46)	<0.001
Sex of household head	Male	10,707	9019	Ref.	
	Female	1369	1694	1.18 (1.07–1.30)	<0.001
Wealth index	Poor	7736	2581	Ref.	
	Middle	2527	2112	1.59 (1.46–1.73)	<0.001
	Rich	1812	6019	2.58 (2.34–2.84)	<0.001
Parity	1 to 4	7059	7819	Ref.	
	5 and more	5017	2894	0.89 (0.78–1.02)	0.1
Evert terminated the pregnancy	No	10,713	9127	Ref.	
	Yes	1363	1585	1.12 (1.01–1.24)	0.03
Wanted to become pregnant	No or later	2304	2267	Ref.	
	Yes	9771	8446	1.02 (0.94–1.12)	0.6
Birth order of the child	First	1684	2345	Ref.	
	2 to 3	3824	4084	0.97 (0.89–1.06)	0.4
	4 to 5	3059	2432	0.92 (0.84–1.05)	0.1
	6 and more	3508	1852	0.87 (0.75–1.02)	0.1
Sex of child	Male	6119	5545	Ref.	
	Female	5956	5168	0.96 (0.90–1.03)	0.3
The birth size of the child	Small	2140	1498	Ref.	
	Average	6056	5460	1.04 (0.96–1.30)	0.4
	Large	3879	3755	1.08 (0.98–1.28)	0.3


Lower-level predictor variables	Categories	Frequency of low-coverage countries		Low coverage countries	
		Suboptimal utilizer	Optimal utilizer	OR (95 % CI)	P -value
Delivery by cesarean section	No	12,005	9891	Ref.	
	Yes	71	822	2.97 (2.30–3.91)	<0.001
Timings of first antenatal care visits	After the first trimester	8249	6185	Ref.	
	In the first trimester	3827	4528	1.20 (1.12–1.29)	<0.001
Women's autonomy in healthcare decision-making	No	6206	4185	Ref.	
	Yes	5870	6528	1.15 (1.07–1.23)	<0.001
Mother occupation	Not working	3207	3470	Ref.	
	Working	8868	7242	0.77 (0.71–0.83)	<0.001
Husband occupation	Not working	1122	684	Ref.	
	Working	10,954	10,028	1.23 (1.08–1.41)	0.002
Husband's education level	No education	5567	2390	Ref.	
	Primary education	3155	2131	1.08 (0.98–1.19)	0.1
	Secondary education	2937	4547	1.09 (0.98–1.21)	0.1
	Higher education	416	1645	1.11 (0.94–1.31)	0.2
Distance to health facility	Not a big problem	6206	7656	Ref.	
	Big problem	5869	3057	0.74 (0.69–0.79)	<0.001
Covered by health insurance	No	11,922	10,155	Ref.	
	Yes	154	558	1.32 (1.03–1.69)	0.03
Media access	No	7227	2806	Ref.	
	Yes	4848	7907	1.61 (1.48–1.72)	<0.001
Family size	Small	3073	3157	1.11 (1.02–1.22)	0.02
	Medium	6935	5913	1.16 (1.03–1.30)	0.02
	Large	2067	1643	Ref.	
DHS-SDG period	Pre SDG	1193	723	Ref.	
	Early SDG	7511	6772	1.56 (1.11–2.19)	0.02
	Action of decades	3372	3217	1.57 (1.08–2.28)	0.02

Our multilevel latent class regression identified a set of individual and household-level determinants that are differentially associated with women's optimal RMNCH service utilization across countries with high and low coverage in sSA. Key determinants influencing women's optimal RMNCH service utilization in both high- and low-coverage settings include maternal education, wealth index, distance to health facilities, media access, and the DHS-SDG period. Conversely, additional predictors, such as maternal age, place of residence, sex of household head, history of pregnancy termination, birth order, birth size, delivery by cesarean section, timing of first antenatal care contact, women's autonomy in healthcare decision-making, maternal occupation, health insurance coverage, and family size, were predominantly associated with optimal utilization in countries with low coverage.

This study revealed that in countries with high coverage, women with secondary education had 1.13 times higher odds of optimal RMNCH service utilization than women with no formal education (AOR: 1.13, 95 % CI: 1.10–1.28). In low coverage countries, women with secondary education had 1.90 times higher odds (AOR: 1.90, 95 % CI: 1.71–2.11), while those with higher education had 3.25 times higher odds (AOR: 3.25, 95 % CI: 2.37–4.46) of optimal utilization relative to women with no formal education. Similarly, the wealth index demonstrated a clear positive gradient. In countries with high coverage, women from middle-income households had 1.18 times the odds, and those from wealthy households had 1.23 times the odds of optimal RMNCH service utilization compared with women from poor households. These associations were stronger in low-coverage countries, where women from middle-income and rich households had 1.59 times (95 % CI: 1.46–1.73) and 2.58 times (95 % CI: 2.34–2.84) higher odds, respectively, than women from poor households.

Women who reported that distance to the nearest health facility was a barrier had lower odds of achieving optimal RMNCH service utilization in high-coverage countries, with an estimated odds ratio of 0.89 (95 % CI: 0.83–0.95) compared with women who did not perceive distance as a barrier. In low-coverage countries, the likelihood of optimal utilization was further reduced by 0.74 among women who reported distance as a barrier (AOR: 0.74, 95 % CI: 0.69–0.790. Women with media access were 1.12 times more likely to achieve optimal RMNCH service utilization in high coverage countries compared to those without media access (AOR: 1.12, 95 % CI: 1.04–1.220), and 1.61 times more likely in low coverage countries (AOR: 1.61, 95 % CI: 1.48–1.72).

Regarding the DHS-SDG survey period, women surveyed during the early SDG period were 1.47 times more likely to achieve optimal RMNCH service utilization in high coverage countries (AOR: 1.47, 95 % CI: 1.12–1.96) and 1.56 times more likely in low coverage countries (AOR: 1.56; 95 % CI: 1.11–2.19) relative to women surveyed in the pre-SDG period. In countries with low coverage, place of residence and timing of the first ANC visit were important predictors of optimal RMNCH service utilization. Women residing in rural areas had lower odds of achieving optimal utilization compared with those in urban areas, with an estimated odds ratio of 0.81 (AOR: 0.81, 95 % CI: 0.74–0.88]. Women who initiated ANC in the first trimester had higher odds of optimal utilization compared with those who began ANC later, with an odds ratio of (AOR: 1.20, 95 % CI: 1.12–1.29].

Inequalities in Optimum RMNCH Service Utilization.

Different socioeconomic and demographic subgroups had different optimal RMNCH service utilization (Table 7). Compared to rural women, urban women used RMNCH services more frequently (absolute difference: 0.23, 95 % CI: 0.22–0.24; ratio: 1.37). Compared to women in the lowest quintile, those in the richest quintile were more likely to attain optimal service usage (0.32, 95 % CI: 0.31–0.33; ratio: 1.58). Higher educated women used RMNCH services more frequently than less educated or uneducated women (0.43, 95 % CI: 0.42–0.44; ratio: 1.79). RMNCH service usage was marginally greater among working mothers than among non-working mothers (0.13, 95 % CI: 0.12–0.14; ratio: 1.12). The use of RMNCH services was concentrated among wealthier and better educated women, according to concentration and slope indices (SII: 0.52 for wealth, 0.47 for education; RII: 2.34 for wealth, 2.12 for education). According to population attributable measures, eliminating wealth-related inequalities might boost national optimum utilization by 0.18 points (25.6 %), and eliminating education-related differences by 0.28 points (40.2 %).

**Table 7:** Socioeconomic and demographic inequalities in RMNCH service utilization.

Equity Dimension	Measure Type	Measure Name	Estimate (95 % CI)	Absolute / Relative
Place of residence	Simple	Difference	0.23 (0.22–0.24)	Absolute
	Simple	Ratio	1.37 (1.36–1.38)	Relative
Maternal occupation	Simple	Difference	0.13 (0.12–0.14)	Absolute
	Simple	Ratio	1.12 (1.08–1.17)	Relative
Wealth index	Disproportionality	Absolute Concentration Index	0.08 (0.07–0.08)	Absolute
	Disproportionality	Relative Concentration Index	0.11 (0.10–0.12)	Relative
	Regression-based	Slope Index of Inequality (SII)	0.52 (0.42–0.61)	Absolute
	Regression-based	Relative Index of Inequality (RII)	2.34 (1.91–2.86)	Relative
	Impact	Population Attributable Risk (PAR)	0.18 (0.17–0.18)	Absolute
	Impact	Population Attributable Fraction (PAF)	25.64 (25.63–25.65)	Relative
Maternal education	Disproportionality	Absolute Concentration Index	0.07 (0.06–0.08)	Absolute
	Disproportionality	Relative Concentration Index	0.10 (0.09–0.12)	Relative
	Regression-based	Slope Index of Inequality (SII)	0.47 (0.39–0.56)	Absolute
	Regression-based	Relative Index of Inequality (RII)	2.12 (1.82–2.48)	Relative
	Impact	Population Attributable Risk (PAR)	0.28 (0.27–0.29)	Absolute
	Impact	Population Attributable Fraction (PAF)	40.24 (40.24–40.26)	Relative

### Sensitivity analysis

At both the individual and cluster levels, sensitivity studies utilizing the maternal care and child care subgroups produced the same two-class result as the complete 12-component RMNCH model. While the subgroup studies verify that the latent class structure is stable and robust across all service domains, the AIC and BIC values for the subgroups were higher than the complete model, indicating that the full set of 12 components offers the most thorough model fit (Table 8).

**Table 8:** Sensitivity analysis of multilevel latent class models using maternal and child service groupings.

Model Specification	$C_{i}$	$M_{j}$	AIC	BIC-Low	BIC-High
Full RMNCH Model	**2**	**2**	**611,377.69**	**621,493.46**	**619,483.96**
Maternal Care Group	2	2	611,937.24	621,939.67	619,939.37
Child Care Group	2	2	611,463.82	621,464.93	619,468.01

Where $C_{i}$ is a lower-level class, and $M_{j}$ is a higher-level class.

## Discussion

This study conducted a thorough multi-country analysis of RMNCH service utilization in 29 sSA countries, employing the MLCA approach to capture heterogeneity at both the individual and country levels. The major goal was to quantitatively classify women's RMNCH service utilization and investigate inequalities in coverage across countries using a data-driven, hierarchical methodology. Model selection using AIC and BIC consistently supported a strong two-tier classification, with two lower-level classes, optimal versus suboptimal utilizers, and two higher-level classes, countries with high versus low RMNCH coverage. The entropy values were extraordinarily high, reaching 1 for higher-level classifications and above acceptable levels for lower-level classes, indicating satisfactory separation and assignment reliability. At the country level, high coverage countries made up 65.6 % of the sample, with 82.7 % of women classified as optimal utilizers. Low coverage countries (34.4 %) had a more fragmented profile, with only 46.5 % optimal utilizers and 53.5 % suboptimal utilizers; examples include Angola, Ethiopia, Guinea, and Madagascar. Individually, 69.5 % of women were optimal utilizers, while only two-thirds of countries had high coverage, revealing access disparities within and between countries. These findings are consistent with prior multi-country studies that have documented ongoing discrepancies in RMNCH service use across sSA, emphasizing the need for targeted, context-specific interventions [34,35].

While this analysis focuses primarily on household- and individual-level determinants of RMNCH service utilization, it is important to acknowledge the absence of key supply-side indicators. Although distance to the nearest health facility was included as a proxy for service access and showed a substantial association with utilization, most supply-side variables—such as facility readiness, staffing adequacy, service availability, and mandatory vaccination policies—were not available across the 29 countries and therefore could not be incorporated into the MLCA model. These unmeasured supply-side constraints may influence service uptake and partly explain the observed between-country differences. Future studies that integrate harmonized health facility–level data with population-based surveys would enable a more complete understanding of both demand- and supply-side determinants of RMNCH service utilization in the region.

The posterior probabilities emphasized the strength of the classification by displaying strong contrasts between the classes. Unlike prior approaches that relied on composite coverage indices (CCI) or arbitrary thresholds [[7], [8], [9], [10], [11]], this method uses the entire range of RMNCH indicators while explicitly accounting for the hierarchical data structure. This provides a more exact and empirically informed classification of both individual and national patterns. The strong agreement observed with studies employing multiple indicators or CCI reinforces the validity of this approach, while its ability to overcome the limitations of index-based or threshold-dependent categorizations positions it as a methodological advancement with significant implications for measuring, monitoring, and addressing inequalities in RMNCH service coverage across sSA.

This study found that countries with a higher agricultural productivity index had higher rates of RMNCH service coverage, which is consistent with earlier studies conducted in India and Africa [36,37]. Higher agricultural output increases household food security, leading to high maternal nutrition, healthier pregnancies, and a higher ability to access important health services [38]. Similarly, countries with high economic capabilities (measured by GDP per capita) were more likely to maintain comprehensive RMNCH coverage. This is consistent with previous research demonstrating that national income permits expenditures in health infrastructure, social protection, and education, all of which improve service accessibility and utilization [20,39]. Economic growth also indirectly promotes maternal service utilization by enhancing female education and empowerment, both of which are important motivators for health-seeking behaviors [40].

In this study, effective governance and higher government health spending were favorably associated with RMNCH coverage, replicating findings from other studies in sSA [41,42]. Functional governments ensure the availability and quality of health services, promote equitable resource distribution, and strengthen accountability [43], whereas adequate funding enhances facility readiness, health worker capacity, and outreach programs, all of which contribute to the continuum of maternal and child care [44]. Furthermore, countries with more gender-equitable legal and economic settings had a larger proportion of women utilizing RMNCH services, supporting previous studies on empowerment and legal protections [45,46]. Gender-equitable policies eliminate sociocultural barriers, boost women's autonomy, and improve access to financial and informational resources, all of which are necessary for consistent use of maternity and child health services [47,48].

In this study, maternal age was associated with differences in optimal RMNCH service utilization. In countries with low coverage, older women (35–49 years) were more likely to use RMNCH services than younger mothers, which is consistent with previous research in West Africa [8], Tanzania [35], and Ethiopia [49]. This pattern is most likely due to accumulated pregnancy experience, a high understanding of maternal and child health concerns, and a higher social position, all of which promote women's autonomy in health-related decision-making [50]. Similarly, maternal education was identified as a critical driver of optimal RMNCH service utilization in both country classes, with low and high coverage. Women with elementary, secondary, or higher education were more likely to use RMNCH services than those without formal education. This conclusion is most likely due to education increasing knowledge, health literacy, and essential awareness of the benefits of expert treatment, allowing women to overcome traditional or financial limitations. Educated women are high able to independently interact with health services on their own, make educated decisions regarding prenatal, birth, and postnatal care, and ensure adherence to the entire RMNCH service continuum [51]. Similarly, children of educated mothers were more likely to benefit from maternal and child health treatments, which is consistent with previous research in sSA and elsewhere [[52], [53], [54]].

In low-coverage countries, as in previous studies [11,55], place of residence contributed to differences in the likelihood of achieving optimal RMNCH service utilization, with rural women receiving fewer services than their urban counterparts. This discrepancy is most likely due to structural hurdles in rural areas, such as higher distances to health facilities, poor transportation infrastructure, and restricted availability of services [56]. Furthermore, gaps in health system decentralization frequently result in reduced service density and quality in rural areas, limiting women's ability to complete the full spectrum of RMNCH treatments [57]. The sex of the household head was linked to variations in service utilization, with children from female-headed households having a higher likelihood of optimal RMNCH service intake, which is consistent with previous research [58]. This could be explained by differences in household goals and resource allocation, as female heads are more likely to exert autonomy and control over maternal and child health expenditures. Female-headed households may also benefit from high social support networks and empowerment dynamics that promote care-seeking behavior [59].

In this study, the household wealth index was identified as a strong predictor of optimal RMNCH service utilization among mothers in both low and high coverage countries. Women from medium and upper-income households were more likely to use the broad spectrum of RMNCH services than those from lower-income households. Wealth presumably lowers financial obstacles to care, such as transportation costs, service fees, and opportunity costs, allowing mothers to receive timely prenatal, delivery, and postnatal care [60]. Higher-income households can also afford preventive and curative treatments, indicating increased health-seeking behavior and the ability to prioritize maternal health requirements [60]. These findings are consistent with previous studies in sSA and other low- and middle-income countries [11,61,62]. Aside from the wealth index, early prenatal care (within the first trimester) was related to a higher likelihood of achieving the RMNCH continuum in both low- and high-coverage countries. These findings are consistent with those of previous studies [11]. Early participation allows for immediate risk assessment, health education, and continuity of treatment, resulting in a healthy trajectory throughout pregnancy and postpartum [63].

The findings showed that women who had previously terminated a pregnancy were more likely to use RMNCH services in countries with low availability of these services. This increased engagement may be due to heightened risk perception and prior experience with adverse pregnancy outcomes, which motivates women to adhere more strictly to prescribed maternal care. Other studies have reported similar findings [10,49]. This result found that women who delivered by cesarean section in countries with low coverage used more RMNCH services. The necessity for institutional care during and after surgery exposes patients to a variety of providers, reflecting both the accessibility and availability of emergency obstetric care [64]. Similar findings have been observed in sSA and other countries [9]. Similarly, maternal autonomy was positively associated with RMNCH service utilization in countries with low coverage. Empowered women are more likely to overcome cultural or familial barriers, exercise decision-making power, and control household resources to obtain care [65]. This is consistent with previous research [45,66]. Employed mothers in countries with restricted coverage were less likely to use RMNCH services, probably because of time constraints and a lack of workplace support, despite the potential financial benefits [67]. Previous studies have revealed similar findings, indicating that competing time demands can impede maternal care-seeking [68,69]. Maternal utilization of RMNCH services was higher among women whose husbands worked, particularly in countries with low coverage. Male employment is likely to increase household income and the ability to afford healthcare, facilitating maternal care seeking [70]. These findings are consistent with prior research that has highlighted the importance of spousal support and economic stability in increasing maternal care utilization [71,72].

In this study, mothers who identified distance as a major barrier were less likely to use RMNCH services in both low- and high-coverage countries. Geographic inaccessibility increases time, cost, and safety costs, particularly in rural sSA, thereby lowering service uptake [73]. This is consistent with a previous study revealing the essential impact of proximity on maternal healthcare access in Ghana [53] and sSA [9]. Mothers with health insurance in countries with low coverage were more likely to use RMNCH services, indicating lower out-of-pocket costs and financial constraints [74]. These findings are consistent with prior research, which found that insurance coverage encourages care-seeking and increases maternal health equity [75]. Media exposure was also favorably associated with RMNCH service utilization in both low- and high-coverage countries. Access to media raises awareness of services, health promotion messaging, and institutional delivery norms, which helps to combat disinformation and traditional barriers. Previous studies have revealed similar patterns [9,11].

Furthermore, moderate and small family sizes were linked to higher RMNCH service utilization in low coverage countries compared to larger families. Smaller households may provide high caregiving assistance and more efficient resource allocation, allowing mothers to access critical services, whereas larger families may encounter financial and logistical obstacles that impede consistent care-seeking [5]. These findings are consistent with previous research on service utilization [76,77]. Surveys conducted during the SDG timeframe showed improvements in maternal RMNCH service utilization in both low and high-coverage countries, indicating the influence of increased global health efforts, policy initiatives, and funding.

The current study shows that different socioeconomic and demographic groups have significantly different optimal RMNCH service use. The strongest determinants were maternal education and household wealth; both absolute and relative measures showed that women with higher levels of education and wealth consistently use more services, which is consistent with evidence from around the world that links structural socioeconomic advantages to better maternal health outcomes [78,79]. Disparities by maternal occupation and between urban and rural areas were lower, but they nevertheless highlight the complexity of injustices. Utilization is consistently higher among socioeconomically advantaged groups, according to concentration indices and regression-based metrics like the slope index of inequality, with the wealthiest and most educated women more than twice as likely to attain optimal service use as the least advantaged [80,81]. According to impact analyses, if all women attained the levels seen in the wealthiest or most educated groups, national coverage might rise by 25–40 % [29,82]. These results demonstrate enduring pro-advantaged disparities and the necessity of focused interventions to remove structural obstacles and increase the coverage of RMNCH services among underprivileged communities throughout the area.

## Conclusion

This study indicates persistent socioeconomic and regional disparities in RMNCH service utilization across sSA, influenced by both individual and national determinants. Using the MLCA, we identified optimal and suboptimal users and countries with high versus low coverage, revealing intricate patterns of service co-utilization. Despite overall progress, women who are poor, undereducated, rural, or who live in countries with poor healthcare systems continue to be disproportionately underserved. To promote equity and accelerate progress towards universal health coverage and the SDGs, these gaps must be addressed through focused, context-specific interventions and multisectoral investments in education, economic empowerment, health infrastructure, and governance.

## Strengths and limitations

The study has a number of advantages. It makes use of nationally representative DHS data and a multi-country scope to enable reliable cross-country comparisons. A sophisticated knowledge of RMNCH service consumption is made possible by the combination of individual and household-level variables with country-level covariates using advanced MLCA. Furthermore, the study offers practical insights for policy and program design by thoroughly examining inequality using both absolute and relative measures. The study does have several drawbacks, though. Its dependence on self-reported service consumption may introduce reporting bias, and its cross-sectional nature restricts the ability to conclude causality. Important supply-side indicators, including facility preparedness, sufficient personnel, service accessibility, and immunization practices, are also absent from the analysis. The observed discrepancies between countries may be partially explained by unmeasured supply-side constraints, even though the distance to the closest health institution was included as a proxy for service access. Additionally, contextual or cultural elements that affect care-seeking behavior were not recorded. Notwithstanding these drawbacks, the results offer vital information to guide focused actions meant to lessen inequalities and enhance RMNCH outcomes throughout sub-Saharan Africa.

## Clinical trial number

Not applicable.

## CRediT authorship contribution statement

**Abebew Aklog Asmare:** Writing – review & editing, Writing – original draft, Visualization, Validation, Supervision, Software, Resources, Project administration, Methodology, Investigation, Formal analysis, Data curation, Conceptualization. **Awoke Seyoum Tegegne:** Writing – review & editing, Visualization, Validation, Supervision, Methodology, Investigation. **Denekew Bitew Belay:** Writing – review & editing, Visualization, Validation, Resources, Methodology, Investigation, Formal analysis, Conceptualization.

## Consent for publication

Not applicable.

## Ethics approval and consent to participate

This research is based on publicly available, anonymous secondary data from the Demographic and Health Surveys (DHS) Program. The DHS data collection techniques were evaluated and approved by the ICF Institutional Review Board (IRB) in the United States and the Department of Health and Human Services' rules for human subject protection (45 CFR 46). All DHS surveys required informed consent, and confidentiality was rigorously protected. Participants under the age of 16 provided parental or guardian consent. We obtained permission to use the data from the DHS Program (https://dhsprogram.com/), and this secondary analysis did not require any additional ethical approval. We confirm that all methods were performed in accordance with the relevant guidelines and regulations and that this study adheres to the ethical principles of the Declaration of Helsinki.

## Funding

This research did not receive any specific grants from funding agencies in the public, commercial, or nonprofit sectors.

## Declaration of competing interest

The authors declare that there are no conflicts of interest among the authors or between the authors and institutions.

## Data Availability

The data used in this study came from the Measure DHS program (https://dhsprogram.com/Data/terms-of-use.cfm) and can be obtained using the protocol indicated in the Methods section. Additional documentation on ethical problems related to the surveys is accessible at http://dhsprogram.com.

## Appendix A Supplementary data

Supplementary material Image 1
