# Supplementary material for: Unveiling hidden heterogeneity and inequalities in the continuum of care for reproductive, maternal, and child health services in sub-Saharan Africa: A multilevel latent class analysis approach
Source: Glob Epidemiol. 2025 Dec 11;11:100237. doi: 10.1016/j.gloepi.2025.100237 (PMC12767714; doi:10.1016/j.gloepi.2025.100237)
Supplement: Supplementary file 1 — Supplementary material [file mmc1.pdf]

```
#+++++++++++++++++++++++++++++++++++++++++++++++++++++++++++++
#++++++++++++++++++++++++++++++++++++++++++++++++++++++++++++++
# Packages used for multilevel latent class analyses
#+++++++++++++++++++++++++++++++++++++++++++++++++++++++++++++
#+++++++++++++++++++++++++++++++++++++++++++++++++++++++++++++
library(multilevLCA)
library(haven)
library(dplyr)
# Orginal Datasets
LCAdata <- read_sav("C://Users//aklog//OneDrive//Documents//Final_LCA_Data.sav")
LCAdata
attach(LCAdata)
names(LCAdata)
dim(LCAdata)
# Items
Antenatal_Care <- as.factor(LCAdata$ANC)
Tetanus_toxoid_injections <- as.factor(LCAdata$TTN)
Facility_delivery <- as.factor(LCAdata$FD)
Skilled_birth_attendance <- as.factor(LCAdata$SBA)
Postnatal_care_mother <- as.factor(LCAdata$PNCM)
Postnatal_care_newborn <- as.factor(LCAdata$PNCN)
BCG_vaccination <- as.factor(LCAdata$BCG)
Polio_vaccination <- as.factor(LCAdata$Polio)
DPT_vaccination <- as.factor(LCAdata$DPT)
Measles_vaccination <- as.factor(LCAdata$Msls)
Age_appropriate_breast_feeding <- as.factor(LCAdata$AABF)
Family_planning <- as.factor(LCAdata$FP)
# independent variables (Individual level independent variables)
Age_mother <- factor(LCAdata$V013,
                     levels = c(0, 1, 2),
                     labels = c("less than 20", "20 to 34", "35 to 49"),
                     ordered = TRUE)
Place_residence <- factor(LCAdata$V025, levels = c(0, 1),
                          labels = c("Urban", "Rural"))
Mother_education <- factor(LCAdata$V106, levels = c(0, 1, 2, 3),
                           labels = c("No education", "Primary education",
                                      "Secondary education", "Higher education"),
                           ordered = TRUE)
Sex_household_head <- factor(LCAdata$V151, levels = c(0, 1),
                             labels = c("Male", "Female"))
Wealth_index <- factor(LCAdata$V190, levels = c(0, 1, 2),
                       labels = c("Poor", "Middle", "Rich"),
                       ordered = TRUE)
Parity <- factor(LCAdata$V201, levels = c(0, 1),
                 labels = c("1 to 4", "5 and more"))
Pregnancy_termination <- factor(LCAdata$V228,levels = c(0, 1),
```

```
                          labels = c("No", "Yes"))
Want_last_child <- factor(LCAdata$V367, levels = c(0, 1),
                    labels = c("No", "Yes"))
Birt_order <- factor(LCAdata$bord_1, levels = c(0, 1, 2, 3),
               labels = c("First", "2 to 3", " 4 to 5", "6 and more"),
               ordered = TRUE)
Sex_child <- factor(LCAdata$B4, levels = c(0, 1),
              labels = c("Male", "Female"))
Birth_size_child <- factor(LCAdata$M18, levels = c(0, 1, 2),
                     labels = c("Small", "Average", "Large"),
                     ordered = TRUE)
Delivery_caesarean_section <- factor(LCAdata$M17, levels = c(0, 1),
                              labels = c("No", "Yes"))
First_ANC_visits <- factor(LCAdata$M13, levels = c(0, 1),
                     labels = c("After first trimster",
                                "At first trimister"))
Women_autonomy <- factor(LCAdata$V743A, levels = c(0, 1),
                   labels = c("No", "Yes"))
Mother_occupation <- factor(LCAdata$V716, levels = c(0, 1),
                      labels = c("Not have occupation",
                                 "Have Occupation"))
Husband_occupation <- factor(LCAdata$V704, levels = c(0, 1),
                       labels = c("Not have occupation",
                                  "Have Occupation"))
Husband_education <- factor(LCAdata$V701, levels = c(0, 1, 2, 3),
                      labels = c("No education", "Primary education",
                                 "Secondary education", "Higher education"),
                      ordered = TRUE)
Health_facility_distance <- factor(LCAdata$V467D, levels = c(0, 1),
                             labels = c("Not a big problem",
                                        "A big problem"))
Health_insurance <- factor(LCAdata$V481, levels = c(0, 1),
                     labels = c("No", "Yes"))
Media_access <- factor(LCAdata$Media, levels = c(0, 1),
                 labels = c("No", "Yes"))
Family_size <- factor(LCAdata$fsize, levels = c(0, 1, 2),
                labels = c("Small", "Medium", "Large"), ordered = TRUE)
SDG_DHS_Year <- factor(
  LCAdata$DHS_Year,
  levels = c(0, 1, 2),
  labels = c("Pre-SDG / Baseline period",
             "Early SDG Implementation period",
             "Decade of Action period")
)
# # independent variables (Higher level independent variables)
Crop_production_index <- as.numeric(LCAdata$Crop_production_index)
```

```
Consumer_price_index <- as.numeric(LCAdata$Consumer_price_index)
Government_Effectiveness <- as.numeric(LCAdata$Government_Effectiveness )
GDP_per_capita <- as.numeric(LCAdata$GDP_per_capita)
Regulatory_Quality <- as.numeric(LCAdata$Regulatory_Quality )
Women_Business_and_the_Law_Index <-
as.numeric(LCAdata$Women_Business_and_the_Law_Index)
Current_health_expenditure <- as.numeric(LCAdata$Current_health_expenditure)
#Prevalence_of_moderate_or_severe_food_insecurity <-
as.numeric(LCAdata$Prevalence_of_moderate_or_severe_food_insecurity)
Voice_and_Accountability <- as.numeric(LCAdata$Voice_and_Accountability)

Country <- factor(LCAdata$Code, levels = c(1,2,3,4,5,6,7,8,9,10,11,12,13,14,15,
                         16,17,18,19,20,21,22,23,24,25,26,27,28, 29),
        labels = c("Angola", "Benin", "Burkina Faso", "Burundi",
             "Cameroon", "Côte d’Ivoire", "Ethiopia", "Gabon",
             "Gambia", "Ghana", "Guinea", "Kenya", "Lesotho",
             "Liberia", "Madagascar", "Malawi", "Mali",
             "Mauritania", "Mozambique", "Nigeria", "Rwanda",
             "Senegal", "Sierra Leone", "South Africa",
             "Tanzania", "Uganda", "Zambia", "Zimbabwe", "Dr. Congo"))
Country

# Create data farmes
wdata <- data.frame(# Items
 Antenatal_Care, Tetanus_toxoid_injections, Facility_delivery,
 Skilled_birth_attendance, Postnatal_care_mother,
 Postnatal_care_newborn, BCG_vaccination, Polio_vaccination,
 DPT_vaccination, Measles_vaccination,
 Age_appropriate_breast_feeding, Family_planning,
 # independent variables (lower level)
 Age_mother, Place_residence, Mother_education,
 Sex_household_head, Wealth_index, Parity, Pregnancy_termination,
 Want_last_child, Birt_order, Sex_child, Birth_size_child,
 Delivery_caesarean_section, First_ANC_visits, Women_autonomy,
 Mother_occupation, Husband_occupation, Husband_education,
 Health_facility_distance, Health_insurance, Media_access,
 Family_size, Country, SDG_DHS_Year,
 # independent variables (higher level)
 Crop_production_index,   Consumer_price_index, Government_Effectiveness,
 GDP_per_capita, Rule_of_Law, Regulatory_Quality,
 Women_Business_and_the_Law_Index,
 Current_health_expenditure, SDG_DHS_Year, Country
)
wdata
#===============================================
# Updated R Code: Horizontal Stacked Bar Plot
```

```r
#=========================================================
# Load necessary libraries
library(dplyr)
library(tidyr)
library(ggplot2)
# Adjust weight
d1 <- Final_LCA_Data
d1 <- d1 %>%
  mutate(weight = V005 / 1000000)

# Select only the binary variables and weight
binary_vars <- c("FP", "TTN", "ANC", "FD", "DPT", "Polio", "Msls",
                 "BCG", "SBA", "AABF", "PNCN", "PNCM")

d1_subset <- d1 %>%
  select(all_of(binary_vars), weight)
# Convert to long format and apply weights
d1_long <- d1_subset %>%
  pivot_longer(cols = all_of(binary_vars), names_to = "variable", values_to = "value") %>%
  group_by(variable, value) %>%
  summarise(weighted_count = sum(weight, na.rm = TRUE), .groups = "drop") %>%
  group_by(variable) %>%
  mutate(percentage = weighted_count / sum(weighted_count) * 100)
# Replace variable names with descriptions
d1_long$variable <- recode(d1_long$variable,
                           FP = "Family Planning",
                           TTN = "Birth Protection Against Neonatal Tetanus",
                           ANC = "Antenatal Care Visits",
                           FD = "Facility Delivery",
                           DPT = "DPT3 Vaccination",
                           Polio = "Polio Vaccination",
                           Msls = "Measles Vaccination",
                           BCG = "BCG Vaccination",
                           SBA = "Skilled Birth Attendance",
                           AABF = "Age-Appropriate Breastfeeding",
                           PNCN = "PNC Within Two Days for Newborn",
                           PNCM = "PNC Within Two Days for Mothers"
)
# Plot Horizontal Stacked Bar Plot
ggplot(d1_long, aes(x = percentage, y = variable, fill = factor(value))) +
  geom_bar(stat = "identity", width = 0.6) +
  scale_fill_manual(values = c("#FFA500", "#2E8B57"),  # Coral red & teal blue
                    name = "Category",
                    labels = c("Not Received", "Received")) +
  labs(x = "Percentage",
       y = "MNCH Services") +
```

```
  geom_text(aes(label = sprintf("%.1f%%", percentage)),
            position = position_stack(vjust = 0.5),
            size = 3.5, color = "black") +
  theme_minimal() +
  theme(axis.text.y = element_text(size = 10),
        axis.title.y = element_text(size = 12, face = "bold"),
        axis.title.x = element_text(size = 12, face = "bold"),
        legend.position = "top")
#+++++++++++++++++++++++++++++++++++++++++++++++++++++++++++++++++++++++++
++++++++
#+++++++++++++++++++++++++++++++++++++++++++++++++++++++++++++++++++++++++
++++++++
#+++++++++++++++++++++++++++++++++++++++++++++++++++++++++++++++++++++++++
++++++++
#   Estimates and Plots Single-Level and Multilevel Latent Class Models
#+++++++++++++++++++++++++++++++++++++++++++++++++++++++++++++++++++++++++
++++++++
#+++++++++++++++++++++++++++++++++++++++++++++++++++++++++++++++++++++++++
++++++++
#+++++++++++++++++++++++++++++++++++++++++++++++++++++++++++++++++++++++++
+++
Y <- c("Antenatal_Care", "Tetanus_toxoid_injections", "Facility_delivery",
       "Skilled_birth_attendance", "Postnatal_care_mother",
       "Postnatal_care_newborn", "BCG_vaccination", "Polio_vaccination",
       "DPT_vaccination", "Measles_vaccination",
       "Age_appropriate_breast_feeding", "Family_planning")
Y
YMatrenal <- c("Antenatal_Care", "Tetanus_toxoid_injections",
               "Facility_delivery", "Skilled_birth_attendance",
               "Postnatal_care_mother", "Postnatal_care_newborn",
               "Family_planning")

YMatrenal
YChild <- c("Age_appropriate_breast_feeding","BCG_vaccination",
        "Polio_vaccination", "DPT_vaccination", "Measles_vaccination")
YChild
id_high <- "Country"
id_high
Z <- c("Age_mother", "Place_residence", "Mother_education",
       "Sex_household_head", "Wealth_index", "Parity", "Pregnancy_termination",
       "Want_last_child", "Birt_order", "Sex_child", "Birth_size_child",
       "Delivery_caesarean_section", "First_ANC_visits", "Women_autonomy",
       "Mother_occupation", "Husband_occupation", "Husband_education",
       "Health_facility_distance", "Health_insurance", "Media_access",
       "Family_size")
Z
```

```
ZH <- c("Crop_production_index", "Consumer_price_index",
        "Government_Effectiveness", "GDP_per_capita", "Regulatory_Quality",
        "Women_Business_and_the_Law_Index", "Current_health_expenditure")
ZH
#++++++++++++++++++++++++++++++++++++++++++++++++++++++++++++++++++++++++
#+++++++++++++++++++++++++++++++++++++++++++++++++++++++++++++++++++++++++
# Sensitivity Analysis
#++++++++++++++++++++++++++++++++++++++++++++++++++++++++++++++++++++++++
#+++++++++++++++++++++++++++++++++++++++++++++++++++++++++++++++++++++++++
M_Mother <- multiLCA(
  data = wdata,
  Y = YMatrenal,
  iT = 2,        # Number of classes at lower level
  id_high = NULL,
  iM = NULL,        # Number of classes at higher level (countries)
  Z = Z,
  Zh = NULL,
  extout = TRUE,
  incomplete = TRUE,
  reord = FALSE,
  verbose = TRUE
)
M_Mother
M_Child <- multiLCA(
  data = wdata,
  Y = YChild,
  iT = 2,        # Number of classes at lower level
  id_high = NULL,
  iM = NULL,        # Number of classes at higher level (countries)
  Z = Z,
  Zh = NULL,
  extout = TRUE,
  incomplete = TRUE,
  reord = FALSE,
  verbose = TRUE
)
M_Child
#+++++++++++++++++++++++++++++++++++++++++++++++++++++++++++++++++++++++
# Multilevel LCA model with independent variables
#++++++++++++++++++++++++++++++++++++++++++++++++++++++++++++++++++++++++++++
F_MLCA_Model <- multiLCA(data = wdata, Y = Y, iT = 2,
                          id_high = id_high, iM = 2,
                    Z = Z, Zh = ZH,  extout = TRUE,
                    incomplete = TRUE,
                    reord = FALSE,
                    verbose = TRUE
```

```
                    )
#++++++++++++++++++++++++++++++++++++++++++++++++++++++++++++++++
# Extracting the outputs based on the model MLCA03
#+++++++++++++++++++++++++++++++++++++++++++++++++++++++++++++++
F_MLCA_Model
# ------------------------------
# --- Posterior matrices to save in SPSS ---
# ------------------------------
cPMX    <- F_MLCA_Model$cPMX
# Posterior joint class assignment for each lower-level unit and higher-level unit
# (proportional assignment based on estimated probabilities)
cPX     <- F_MLCA_Model$cPX
# Posterior lower-level class assignment given high-level class membership
# (proportional assignment; conditional probabilities for lower-level units)
mSumPX  <- F_MLCA_Model$mSumPX
# Posterior higher-level class assignment for lower-level units after marginalization over lower-
level classes
# (proportional assignment; sums over lower-level class probabilities)
mPW     <- F_MLCA_Model$mPW
# Posterior higher-level class assignment for higher-level units
# (proportional assignment; probability of each higher-level latent class)
mPW_N   <- F_MLCA_Model$mPW_N
# Posterior higher-level class assignment for lower-level units
# (proportional assignment; conditional probabilities for lower-level units at the higher-level)
mPMsumX <- F_MLCA_Model$mPMsumX
# Posterior lower-level class assignment for lower-level units after marginalization over higher-
level classes
# (proportional assignment; sums over higher-level latent classes)
# ------------------------------
# Convert matrices to data frames for SPSS
# ------------------------------
df_cPMX    <- as.data.frame(cPMX)
df_cPX     <- as.data.frame(cPX)
df_mSumPX  <- as.data.frame(mSumPX)
df_mPW     <- as.data.frame(mPW)
df_mPW_N   <- as.data.frame(mPW_N)
df_mPMsumX <- as.data.frame(mPMsumX)
# Optional: add ID column for row tracking
df_cPMX$ID    <- 1:nrow(df_cPMX)
df_cPX$ID     <- 1:nrow(df_cPX)
df_mSumPX$ID  <- 1:nrow(df_mSumPX)
df_mPW$ID     <- 1:nrow(df_mPW)
df_mPW_N$ID   <- 1:nrow(df_mPW_N)
df_mPMsumX$ID <- 1:nrow(df_mPMsumX)
# ------------------------------
# Save posterior matrices as SPSS .sav files
```

```
# ------------------------------
write_sav(df_cPMX,    "cPMX.sav")
write_sav(df_cPX,     "cPX.sav")
write_sav(df_mSumPX,  "mSumPX.sav")
write_sav(df_mPW,     "mPW.sav")
write_sav(df_mPW_N,   "mPW_N.sav")
write_sav(df_mPMsumX, "mPMsumX.sav")
# ------------------------------
# Other model components (keep in positions)
# ------------------------------
F_MLCA_Model$vOmega       # Higher-level class proportions given the covariates
F_MLCA_Model$mPi          # Lower-level class proportions given the higher-level latent classes
and the covariates
F_MLCA_Model$mPi_avg      # Sample average of mPi
F_MLCA_Model$mPhi         # Response probabilities given the lower-level latent classes
F_MLCA_Model$vAlpha       # Intercept and slope parameters in logistic models for conditional
higher-level class membership
F_MLCA_Model$cGamma       # Intercept and slope parameters in logistic models for
conditional lower-level class membership
F_MLCA_Model$mBeta        # Intercepts in logistic parametrization for response probabilities
F_MLCA_Model$parvec       # Vector of logistic parameters
F_MLCA_Model$SEs_unc      # Uncorrected standard errors
F_MLCA_Model$SEs_cor      # Corrected standard errors
F_MLCA_Model$SEs_cor_alpha  # Corrected standard errors only for the alphas
F_MLCA_Model$SEs_cor_gamma  # Corrected standard errors only for the gammas
F_MLCA_Model$mQ           # Cross-derivatives for asymptotic standard error correction in two-
step estimation
F_MLCA_Model$Varmat_unc   # Uncorrected variance-covariance matrix
F_MLCA_Model$Varmat_cor   # Corrected variance-covariance matrix
F_MLCA_Model$Infomat      # Expected information matrix
F_MLCA_Model$cAlpha_Info  # Expected information matrix only for the alphas
F_MLCA_Model$cGamma_Info  # Expected information matrix only for the gammas
F_MLCA_Model$mV2          # Inverse of information matrix for structural model
F_MLCA_Model$LLKSeries    # Full log-likelihood series for EM algorithm
F_MLCA_Model$vLLK         # Current log-likelihood for higher-level units
F_MLCA_Model$mScore       # Contributions to log-likelihood score
F_MLCA_Model$mAlpha_Score # Contributions to log-likelihood score only for the alphas
F_MLCA_Model$mGamma_Score # Contributions to log-likelihood score only for the gammas
# ------------------------------
# Plot lower-level item probabilities
# ------------------------------
plot(F_MLCA_Model)  # plots item probabilities for lower-level classes
#Plotting posterior probabilities for RMNCH services
# Load libraries
library(ggplot2)
library(dplyr)
```

```
library(tidyr)
# Data: posterior probabilities
rmnch_probs <- data.frame(
  Service = c("Antenatal care", "Tetanus toxoid", "Facility delivery",
            "Skilled birth attendance", "Postnatal care (mother)",
            "Postnatal care (newborn)", "BCG vaccination", "Polio vaccination",
            "DPT3 vaccination", "Measles vaccination",
            "Age-appropriate breastfeeding", "Family Planning"),
  Optimal = c(0.2471, 0.5266, 0.9705, 0.9797, 0.7317, 0.7556,
            0.9722, 0.7679, 0.8666, 0.8559, 0.6592, 0.4103),
  Suboptimal = c(0.1200, 0.3497, 0.1954, 0.2465, 0.0990, 0.0918,
              0.6030, 0.3844, 0.3945, 0.4385, 0.6285, 0.1463)
)
# Reshape for plotting
rmnch_long <- rmnch_probs %>%
  pivot_longer(cols = c("Optimal", "Suboptimal"),
             names_to = "Class",
             values_to = "Probability")

# Order services in continuum of care sequence
rmnch_long$Service <- factor(rmnch_long$Service,
                          levels = c("Antenatal care", "Tetanus toxoid", "Facility delivery",
                                 "Skilled birth attendance", "Postnatal care (mother)",
                                 "Postnatal care (newborn)", "BCG vaccination", "Polio vaccination",
                                 "DPT3 vaccination", "Measles vaccination",
                                 "Age-appropriate breastfeeding", "Family Planning")
)

# Plot
ggplot(rmnch_long, aes(x = Service, y = Probability, fill = Class)) +
  geom_col(position = position_dodge(width = 0.7), width = 0.65) +
  geom_text(aes(label = round(Probability, 2)),
          position = position_dodge(width = 0.7),
          vjust = -0.3, size = 3) +
  scale_fill_manual(values = c("Optimal" = "#1f78b4", "Suboptimal" = "#ff7f00")) +
  scale_y_continuous(limits = c(0, 1), expand = c(0, 0)) +
  labs(x = "RMNCH Service Components", y = "Posterior Probability",
     fill = "Latent Class") +
  theme_minimal(base_size = 12) +
  theme(axis.text.x = element_text(angle = 45, hjust = 1),
      legend.position = "top",
      panel.grid.major.x = element_blank())
# With line
# Load libraries
library(ggplot2)
library(dplyr)
```

```
library(tidyr)
# Data: posterior probabilities
rmnch_probs <- data.frame(
  Service = c("Antenatal care", "Tetanus toxoid", "Facility delivery",
              "Skilled birth attendance", "Postnatal care (mother)",
              "Postnatal care (newborn)", "BCG vaccination", "Polio vaccination",
              "DPT3 vaccination", "Measles vaccination",
              "Age-appropriate breastfeeding", "Family Planning"),
  Optimal = c(0.2471, 0.5266, 0.9705, 0.9797, 0.7317, 0.7556,
              0.9722, 0.7679, 0.8666, 0.8559, 0.6592, 0.4103),
  Suboptimal = c(0.1200, 0.3497, 0.1954, 0.2465, 0.0990, 0.0918,
                 0.6030, 0.3844, 0.3945, 0.4385, 0.6285, 0.1463)
)
# Reshape for plotting
rmnch_long <- rmnch_probs %>%
  pivot_longer(cols = c("Optimal", "Suboptimal"),
               names_to = "Class",
               values_to = "Probability")
# Order services in continuum of care sequence
rmnch_long$Service <- factor(rmnch_long$Service,
                             levels = c("Antenatal care", "Tetanus toxoid", "Facility delivery",
                                        "Skilled birth attendance", "Postnatal care (mother)",
                                        "Postnatal care (newborn)", "BCG vaccination", "Polio vaccination",
                                        "DPT3 vaccination", "Measles vaccination",
                                        "Age-appropriate breastfeeding", "Family Planning")
)
# Line plot
ggplot(rmnch_long, aes(x = Service, y = Probability, group = Class, color = Class)) +
  geom_line(size = 1.2) +
  geom_point(size = 3) +
  geom_text(aes(label = round(Probability, 2)),
            vjust = -0.8, size = 3) +
  scale_color_manual(values = c("Optimal" = "#1f78b4", "Suboptimal" = "#ff7f00")) +
  scale_y_continuous(limits = c(0, 1), expand = c(0, 0)) +
  labs(x = "RMNCH Service Components", y = "Posterior Probability",
       color = "Latent Class") +
  theme_minimal(base_size = 12) +
  theme(axis.text.x = element_text(angle = 45, hjust = 1),
        legend.position = "top",
        panel.grid.major.x = element_blank())
```
